# Closing the gaps in opioid use disorder research, policy and practice: conference proceedings

**DOI:** 10.1186/s13722-018-0123-3

**Published:** 2018-11-13

**Authors:** Matthew A. Miclette, Jared A. Leff, Isabella Cuan, Jeffrey H. Samet, Brendan Saloner, Gary Mendell, Yuhua Bao, Michael A. Ashburn, Marcus A. Bachhuber, Bruce R. Schackman, Daniel E. Polsky, Zachary F. Meisel

**Affiliations:** 1 Leonard Davis Institute of Health Economics, 3641 Locust Walk, Room 310, Philadelphia, PA 19104 USA; 2 Weill Cornell Medical College, 425 East 61st Street, Suite 301, New York, NY 10065 USA; 3 University of Pennsylvania, 3451 Walnut Street, Philadelphia, PA 19104 USA; 4 Boston University School of Medicine, 72 East Concord Street, Boston, MA 02118 USA; 5 Johns Hopkins Bloomberg School of Public Health, 615 N. Wolfe Street, Baltimore, MD 21205 USA; 6 Shatterproof, 950 Sixth Ave, 10th Floor, New York, NY 10001 USA; 7 Perelman School of Medicine, University of Pennsylvania, 3400 Civic Center Blvd, Philadelphia, PA 19104 USA; 8 Montefiore Medical Center/Albert Einstein College of Medicine, 1300 Morris Park Avenue, Bronx, NY 10461 USA

**Keywords:** Opioid use disorder, Drug policy, Opioid policy, Knowledge broker

## Abstract

Drug overdose deaths involving opioids have surged in recent years and the economic cost of the opioid epidemic is estimated to be over $500 billion annually. In the midst of calls for declaring a national emergency, health policy decision makers are considering the best ways to allocate resources to curb the epidemic. On June 9, 2017, 116 invited health researchers, clinicians, policymakers, health system leaders, and other stakeholders met at the University of Pennsylvania to discuss approaches to address the gaps in evidence-based substance use disorder policy and practice, with an emphasis on the opioid epidemic. The conference was sponsored by the Center for Health Economics of Treatment Interventions for Substance Use Disorder, HCV, and HIV (CHERISH), a NIDA-funded National Center of Excellence, and hosted by the Leonard Davis Institute of Health Economics of the University of Pennsylvania. The conference aims were to: (1) foster new relationships between researchers and policymakers through a collaborative work process and (2) generate evidence-based policy recommendations to address the opioid epidemic. The conference concluded with an interactive work session during which attendees self-identified as researchers or policymakers and were divided equally among 13 tables. These groups met to develop and present policy recommendations based on an opioid use disorder case study. Thirteen policy recommendations emerged across four themes: (1) quality of treatment, (2) continuity of care, (3) opioid prescribing and pain management, and (4) consumer engagement. This conference serves as a proposed model to develop equitable, working relationships among researchers, clinicians, and policymakers.

## Introduction

An estimated 20 million people in the United States meet the criteria for substance use disorder (SUD) [1]. In 2015, the opioid epidemic alone had an estimated economic cost of 504 billion USD when taking into account healthcare, criminal justice, and loss of productivity [2]. The Centers for Disease Control and Prevention (CDC) estimate more than 63,600 drug overdose deaths occurred in 2016—a 21% increase from 2015 [3]. The President’s Commission on Combating Drug Addiction and the Opioid Crisis recommended declaring the epidemic a national emergency in their final report in November 2017 [4] and the National Institutes of Health (NIH) HEAL initiative (Helping to End Addiction Long-term) launched in June 2018 to provide scientific solutions to the national opioid overdose epidemic, including improved treatment strategies for pain and opioid use disorder (OUD) [5].

The 2016–2020 National Institute on Drug Abuse (NIDA) Strategic Plan highlights a significant research-to-practice gap in prevention and treatment strategies for SUD. Many effective interventions have been identified by research scientists, but remain underutilized [6]. While medical professionals frequently rely on evidence-based practices, research-informed decisions are less common among policymakers. Instead, policymakers often rely on intuition, ideology, or conventional wisdom [7]. Policymakers most often credit in-person relationships with researchers as the most influential factor in determining the use of research evidence [8]. Early engagement with policymakers may help researchers identify meaningful research questions and encourage researchers to become a part of the translation from evidence to policy and practice [9].

The Center for Health Economics of Treatment Interventions for Substance Use Disorder, HCV, and HIV (CHERISH) is a multi-institutional Center of Excellence, funded by NIDA [10]. The Center’s mission is to develop and disseminate health economic research on healthcare utilization, health outcomes, and health-related behaviors that informs SUD treatment policy, and HCV and HIV care of people who use substances. To increase the impact of this research, CHERISH supports researchers in addressing the needs of integrated healthcare system providers and payers. The Center is a collaboration among Weill Cornell Medicine, Boston Medical Center, the University of Pennsylvania, and the University of Miami Miller School of Medicine. The Dissemination and Policy Core, located at the Leonard Davis Institute of Health Economics, University of Pennsylvania, supports CHERISH researchers in employing dissemination practices to increase visibility and impact of research among policymakers and the public.

## Purpose

The “Substance Use Disorder in America: Research to Practice, and Back Again” conference convened policymakers, who make decisions about, and researchers, who study the treatment of SUD in the United States. Relationships between policymakers and researchers is a key theme that the conference sought to address. The first aim of the conference was to develop new relationships between researchers and policymakers through collaborative work. The second aim was to generate a list of policy recommendations to address the opioid epidemic.

## Conference overview

On June 9, 2017, 116 invited health researchers, clinicians, policymakers, health system leaders, and other stakeholders met at the University of Pennsylvania. There were three conference breakout sessions that covered: (1) opioid prescribing, (2) evidence-based treatment of OUD, and (3) the integration of SUD treatments into the United States healthcare system. The first 45 min of breakout sessions were led by policymakers. After a 15-min networking break, researchers then led the second 45-min session (Table 1). After the breakout groups, the keynote, Former Congressman Patrick Kennedy, shared his political pursuit of insurance coverage parity for mental health and SUD treatment [11]. The day concluded with an interactive work session focused on exchanging policy ideas about solutions to curb the opioid epidemic. Detailed content from sessions and speakers can be found in the full conference proceedings [12]; this manuscript focuses on interactive work session.

**Table 1:** Conference sessions and speakers

Session theme	Policymakers	Researchers
Opening Plenary: Lessons Learned from Distinguished Careers	Richard G. Frank, PhD Harvard University Joshua Sharfstein, MD Johns Hopkins University	Dan Polsky, PhD (Moderator) Leonard Davis Institute of Health Economics A. Thomas McLellan, PhD Treatment Research Institute
Health Systems: Diagnosis and Treatment	Jeffery Samet, MD (Moderator) Boston University Hillary Kunins, MD New York City Department of Health and Mental Hygiene Colleen LaBelle, MSN, RN-BC Boston University Jack Stein, PhD National Institute on Drug Abuse	Marcus Bachhuber, MD (Moderator) Albert Einstein College of Medicine Kathy Bradley, MD University of Washington Adam Gordon, MD University of Utah Jennifer McNeely, MD New York University
Opioid Prescribing: Striking a Balance	Michael Ashburn, MD (Moderator) University of Pennsylvania Jean Bennett, PhD Substance Abuse and Mental Health Services Administration Rachel Levine, MD Pennsylvania Physician General Rita Noonan, PhD Centers for Disease Control and Prevention	Yuhua Bao, PhD (Moderator) Weil Cornell, Moderator Deborah Dowell, MD, MPH Centers for Disease Control and Prevention Dan Hartung, PharmD Oregon State University Stefan Kertesz, MD University of Alabama at Birmingham
Treatment: Access, Coverage, Quality, Costs	Gary Mendell, MBA (Moderator) Shatterproof A. Thomas McLellan, PhD Treatment Research Institute John O’Brien, MS Technical Assistance Collaborative	Brendan Saloner, PhD (Moderator) Johns Hopkins University Richard Frank, PhD Harvard University Tami Mark, PhD Research Triangle Institute International Harold Pollack, PhD University of Chicago

## Bridging the gap: Methods

The conference concluded with an interactive work session focused on the exchange of ideas and solutions to curb the opioid epidemic. Attendees were asked to self-identify as a researcher or policymaker and sit at color-coded seat assignments designed to distribute researchers and policymakers equally among the 13 tables at the conference.

Participants were shown a short video vignette case study featuring a woman sharing her daughter’s struggles with SUD and events leading to her ultimate opioid overdose death. The daughter was referred to multiple inpatient treatment programs by their health insurance provider, during which medication for OUD was discontinued, and post treatment follow-up care was not coordinated. The video was developed at the University of Pennsylvania as a part of a Patient-Centered Outcomes Research Institute (PCORI) funded study on comparative effectiveness of opioid risk communication strategies for pain management (NCT03134092) and is not yet publicly available as of October 2018.

Each group of 5–8 participants were given 45 min to develop policy recommendations to address the many complexities posed by this case. The groups shared their top ideas with the larger conference.

Two members of the conference planning staff (MAM, IC) documented in real-time the discussion on policy recommendations from each of the 13 groups; audio recordings were also made and available for later reference. Following the conference, the conference planning staff members consolidated notes to create a single list of all policy recommendations made during the session.

## Bridging the gap: Results

A subgroup of four authors (MAM, ZFM, DEP, BRS) synthesized the list of policies into four emerging themes by the policy intersection with delivery of care: (1) quality of treatment, (2) continuity of care, (3) opioid prescribing and pain management, and (4) consumer engagement. Examples of how these policies might be implemented are provided by the authors and included in the descriptions below.

### Quality of treatment

#### Tie insurer payment to minimum standards for treatment based on evidence-based practice and continuity of care

No single treatment is the best fit for all individuals with OUD. However, medications for OUD are associated with a host of benefits [13], including reducing the risk of death after opioid overdose, yet remain underutilized [14]. Programs that restrict or discontinue medications for OUD (“abstinence only” programs) should be subject to evaluation prior to payment by insurers. Treatments should have proven efficacy as to not render unnecessary harm.

#### Eliminate or reduce the burden of regulations on buprenorphine prescribing

Under current regulations, qualified providers must apply for a Drug Enforcement Agency waiver submitted through the Substance Abuse Mental Health Service Administration to prescribe buprenorphine. This requires an 8-h course at minimum. After receiving a waiver, providers are restricted to concurrently treating up to 30 patients with OUD. Physicians may submit additional waiver request to increase this limit to 100 patients after 1 year and 275 patients after 2 years [15]. Buprenorphine prescribing is no more complicated or dangerous than treatments regularly provided by primary care providers [16]. Many believe that the waver is an unnecessary bureaucratic burden. Options suggested include reducing the time length of the training course, eliminating waver course fees, and a federal review to determine if provider limits on the number of patients who are eligible to receive buprenorphine restrict overall access to treatment.

#### Create an independent accreditation body that provides a complete listing of available treatment centers and quality scores for treatment facilities

This proposed accreditation body would hold treatment centers accountable by publicly reporting quality metrics, including individual case management and adherence to treatment guidelines. Treatment center scores and contact information may improve customer knowledge and access to quality care. Additionally, the quality scores could be tied to insurance payments.

### Continuity of care

#### Assure in-person or telephone care coordination following discharge after a nonfatal overdose, through peer support or providers who prescribe medications for OUD

Effective case management is necessary to coordinate medical and mental health services of individuals with OUD. After a nonfatal opioid overdose, individuals have an increased risk of death, not from just drug overdose, but a range of mental and physical health conditions within 12 months [17]. Peer support studies have demonstrated reduced relapse rates and increased treatment retention [18]. Our recommendation is for peer support availability at multiple touch points, such as emergency and inpatient units and harm reduction organizations.

#### Promote the hub and spoke models to ensure primary care physicians feel comfortable and supported prescribing medications for OUD

The aim of hub and spoke models are to increase access to buprenorphine treatment by increasing the total number of prescribers. Primary care providers—along with other qualified prescribers—serve as the “spokes” connected to a central “hub.” The hub consists of OUD treatment specialist. The experts at the hubs can help initially stabilize patients and provide ongoing consultation to the spokes. There are multiple hub and spoke models, including the Vermont Hub and Spoke Model and Project ECHO [19]. The development and implementation of the hub and spoke models have contributed to increased access to medications for OUD in Vermont, a rural state that offered no medications for OUD prior to 2000 [20]. There are several US states developing and implementing the hub and spoke model. Researchers should assist in the development, implementation, and evaluation of state hub and spoke treatment systems.

#### Promote emergency department (ED) induction of buprenorphine prior to discharge or hospital admission

Initiating buprenorphine induction in the ED serves as an opportunity to engage patients in treatment. Buprenorphine treatment initiated in the ED significantly increased OUD treatment engagement and reduced self-reported illicit opioid use in a random controlled trial, compared to brief intervention and referral alone [21]. Current research is underway evaluating how to best implement and scale up approaches to encourage ED induction [22].

### Opioid prescribing/pain management

#### Require insurance companies to cover alternative pain treatment modalities, so that opioids are not the default pain management

Despite significant increases in opioid prescribing in the 2000s, the prevalence of pain has remained consistent. Moreover, non-opioid treatments, such as nonsteroidal anti-inflammatory drugs (NSAIDs), decreased in ED visits [23]. This mandate would expand effective pain treatment alternatives to opioid therapy, including pharmacologic (e.g. anticonvulsant class) and nonpharmacologic options (e.g. physical therapy).

#### Tie the use and development of prescription guidelines to federal funding

This proposal underscores the need for healthcare facilities to be held to a standard of care. Facilities should implement and promote opioid prescribing guidelines, such as the CDC Guideline for Prescribing Opioids for Chronic Pain [24].

#### Develop a state scorecard on prescribing that ranks the states in relation to their goals

The proposed scorecard would attempt to hold state government leaders accountable to their constituents, against their own performance measures. The opioid epidemic is becoming an increasingly important nonpartisan issue across the country [25]. The group discussed a wide variety of national options including number of waivered providers per capita and state-specific measures—such as changes in insurance regulations designed to reduce barriers to evidence-based treatments.

### Consumer engagement

#### Create a centralized system of treatment facilities and providers where patients can sign up themselves (“Airbnb”-type model)

This recommendation was presented as an opportunity to enhance availability of treatment on demand. Over 90% of adults between 18 and 49 years old have a smartphone in the United States [26]. A mobile application and website could be established similar to Airbnb—an online platform to reserve short-term and vacation housing rentals. Treatment facilities could use this online platform to update and project inpatient and outpatient treatment availability, and consumers could enter treatment as soon as it becomes available. A standard scheduling system could further improve treatment coordination across health systems.

#### Develop a family and consumer social marketing campaign

There are multi-million dollar state investments in social marketing for the opioid epidemic [27]. This proposal suggests building health communication campaigns that increase public awareness of the risk associated with prescription opioids and to reduce the negative public stigma associated with OUD. The NIDA strategic plan describes social media as an opportunity to provide innovative prevention interventions. Social marketing campaigns should be developed using health communication theory and guided by an established framework, such as the CDC’s Health Communicator’s Social Media Toolkit [28].

#### Produce consumer-driven rating system or recorded metric of treatment programs

A commonly used consumer-driven online rating system is Yelp. On the Yelp website, consumers can provide quantitative (e.g. star rating) and qualitative (e.g. Yelp review) metrics of dining establishments. This recommendation is to develop a similar platform to provide consumer feedback on patient experience with inpatient and outpatient OUD treatment programs. Further discussion and evaluations are necessary to develop, managed, and sustain this type of system.

#### Fund programs that incentivize individuals into treatment (and start with research on best incentive programs)

Incentive programs attempt to engage patients in treatment and recovery by providing rewards or otherwise motivating patients. Programs should review previous SUD motivational incentives, such as those studied in the NIDA clinical trials network [29].

## Discussion

There is a significant research-to-practice gap in the implementation of evidence-based prevention and treatment strategies [6]. Policymakers have identified quality and trust as critical elements to sustained relationships with researchers and the use of research evidence, but several barriers exist. There are few incentives in academia for researchers to engage directly with policymakers. Additionally, researchers may take years to complete a study that includes a complex methodology, while policymakers often require quick options, that have perceived relevance and clear methods they understand [30]. Research organizations, such as CHERISH, are in a unique position to close this gap through knowledge transfer between their research affiliates and key stakeholders.

The “Substance Use Disorder in America: Research to Practice, and Back Again” conference is a model to develop equitable, working relationships between researchers and policymakers. Three breakout sessions were led by policymakers and researchers. Attendees collaborated in mixed groups of researchers and policymakers on a case study which resulted in a list of policy recommendations generated by a diverse group of national experts in SUD. These findings should be further investigated for feasibility in scale-up to curb the opioid epidemic. Conference attendees were also encouraged to stay engaged with individuals they met at the conference and others outside their professional field in order to continue the work toward the solutions discussed during the conference.

The conference achieved the Center’s short-term objective of developing new relationships between researchers and policymakers through collaborative work. Ultimately, long-term success will depend on the forged connections between researchers and policymakers. Research organizations, such as CHERISH, have an opportunity to foster these linkages further. Research organizations should look for opportunities to engage their research affiliates with policymakers that are interested in SUD policy, particularly early in the process to generate meaningful research questions and forge a lasting working relationship.

## Abbreviations

CHERISH: Center for Health Economics of Treatment Interventions for Substance Use Disorder, HCV, and HIV
LDI: Leonard Davis Institute of Health Economics
SUD: substance use disorder
OUD: opioid use disorder
PDMP: prescription drug monitoring program
CDC: Centers for Disease Control
NIDA: National Institute on Drug Abuse
HHS: Department of Health and Human Services
NCHS: National Center for Health Statistics
